# The Power of Plagues

**DOI:** 10.3201/eid1210.060803

**Published:** 2006-10

**Authors:** José G. Rigau-Pérez

**Affiliations:** * Universidad de Puerto Rico, Río Piedras, Puerto Rico, USA

**Keywords:** The Power of Plagues, Irwin Sherman, Book Review, review

The purpose of this book is to make the science of epidemic diseases accessible and understandable; to guide the general reader through the maze of contagious diseases, their past importance, the means by which we came to understand them, and how they may affect our future. This commentary on general and disease-specific concerns covers the nature of plagues; plagues, the price of being sedentary (an evolutionary view); 6 plagues of antiquity (urinary schistosomiasis, the plague of Athens, malaria in Rome, the Antonine plague, the plagues of Cyprian and Justinian); bubonic plague; AIDS (including a history of virology and an account of leukocyte function); typhus; malaria (plus an explanation of sickle cell disease and genetics); cholera; smallpox; preventing plagues (the immune system, with a coda on vaccine development); the plague protectors (antisepsis and antimicrobial drugs); syphilis; tuberculosis; leprosy; 6 plagues of Africa (sleeping sickness, river blindness, guinea worm, yellow fever, malaria, and hookworm) with the history of exploration and exploitation of this continent; plagues without germs (pellagra, beriberi, scurvy, and rickets); and emerging plagues (rodentborne, West Nile virus, bovine spongiform encephalopathy and Creutzfeldt-Jakob disease, and influenza). The text covers not only the geography, history, microbiology, and physiology of these infections but also their influence on plastic art, movies, literature, and music (with a special fondness for nursery rhymes).

Surprisingly, the role of contemporary epidemiologic methods and governmental institutions is not examined. No explanation is included of how present-day public health officials go about detecting a problem, how they define an epidemic, how they use data such as incidence or attack rates to identify the cause, and how laws and regulations (e.g., vaccine requirements for school entry and rules for production of food and biological materials) are indispensable for disease prevention. The text would have profited from another round of editing to modify overly forceful generalizations, tighten the discussion, and check for historical and medical accuracy. For example, acyclovir is not AZT, and AZT was not available for first-line treatment of AIDS in the early 1980s; cholera is not slowly creeping into the Western Hemisphere, but it produced large epidemics in Central and South America in the 1990s; Figure 9.7B is not an antivaccination statement from Boston in 1902 but, as the engraving itself indicates, a provaccine statement from England in 1898; vaccination with *Mycobacterium bovis* BCG does not cause the tuberculin test result to be negative; and malaria control efforts in the United States were not interrupted by World War II but, on the contrary, were enhanced by the creation of an agency called Malaria Control in War Areas.

This is a concise and clear account of the biologic and historical determinants of epidemic diseases. It is marred by a small number of factual errors and a failure to include epidemiologic and public health methods as components of the equation that determines the power of plagues.

